# Prevalence and correlates of perceived age-related discrimination among older adults in India

**DOI:** 10.1186/s12889-022-13002-5

**Published:** 2022-03-22

**Authors:** Priya Maurya, Palak Sharma, T. Muhammad

**Affiliations:** International Institute for Population Sciences, Mumbai, Maharashtra 400088 India

**Keywords:** Perceived age discrimination, Socioeconomic, Health status, Older adult

## Abstract

**Background:**

Age is one of the predominant reasons for perceived discrimination in developing world where older people are considered a non-contributing burden. The present study explores the prevalence and correlates of perceived age discrimination among older Indian adults.

**Methods:**

A cross-sectional study was conducted using a large representative survey data from the Longitudinal Ageing Study in India conducted during 2017–18. Participants included 31,464 older adults aged 60 years and above. Multivariable logistic regression analysis was used to test the associations between selected background characteristics and perceived age discrimination.

**Results:**

The analysis revealed that 10.33 percent of older adults perceived their age as the main reason for discrimination, which was 11.86% among the oldest-old. Older adults with more than 10 years of schooling were 32% [adjusted odds ratio (AOR): 0.68; confidence interval (CI): 0.51—0.89] less likely to perceive age discrimination compared to their uneducated counterparts. The odds of perceived age discrimination were higher among older adults who earlier worked [AOR: 1.73; CI: 1.46—2.05] and currently working [AOR: 1.61; CI: 1.31—1.96] as compared to those who never worked. Further, having difficulty in instrumental activities of daily living (IADL) [AOR: 1.43; CI: 1.25 -1.65] and having one chronic condition [AOR: 1.16; CI: 1.02 -1.34] were associated with increased odds of perceived age discrimination among older adults.

**Conclusion:**

Older adults with lower socioeconomic status, currently working, having more chronic conditions, difficulty in IADL and belonging to rural areas were found to perceive higher age discrimination than their counterparts. The findings of the study have important implications for policy makers with respect to strategies such as making the vulnerable populations aware of their legal rights that help in the prevention of age-based discrimination in the country.

## Background

Population ageing is an ongoing demographic phenomenon globally due to a significant increase in life expectancy coupled with the decline in fertility and improved mortality conditions in recent decades [1]. According to the United Nations ageing report (2017), the total older population (60 years and above) was 962 million in 2017 worldwide and the estimate indicates that the older population could grow to around 2.1 billion by 2050. This shift in age structure is affected by social and economic changes in any society experiencing ageing in the twenty-first century [2]. The transition itself has a keen impact on social, economic and health conditions for the individual, family, and society, such as intergenerational social support system, pension, retirement, and other social benefits [3, 4]. Health and associated medical cost in older age is another issue since older people tend to be more vulnerable to chronic conditions, resulting in negative attitudes in old age. Consequently, the demographic transition is now a significant challenge at the global level [5]. With this emerging phenomenon, ageism and discrimination arise as significant social issues [6].

Perceived discrimination is defined as an individual’s perception of unfairly treating a person or group based on certain characteristics that they possess, such as age, gender, caste, religion, financial status or physical appearance [7, 8]. It is evident from previous studies that more negative views on ageing are related to an increased risk of perceiving age discrimination. The term ageism was first introduced by Butler in 1969 and defined as “systematic stereotyping of and discrimination against people because they are old” [9]. Thus, ageism is often associated with negative attitudes towards older people [10].

Previous studies indicate that age and gender are the predominant reasons for perceived discrimination in the developing world, where older people are considered a non-contributing burden [11, 12]. Perceived discrimination because of one’s age has the highest prevalence as compared to other reasons for discrimination [13, 14]. It is evident that the perceived age discrimination is likely to increase with age and its peak has been noted around the age of 50 years or at the pre-retirement age [6, 15, 16]. Perceived age discrimination is highly related to various other factors like gender, education, lower levels of household wealth, marital status and respondents’ current employment status [17, 18]. Gendered ageism covers the inter-sectionality of age and gender bias. It is evident that ageism hits women earlier and harder [19]. Perceived age discrimination is highly associated with poor self-rated health [16, 18, 20, 21], and higher psychological distress/poor mental health [21–24]. Similarly, studies have shown that negative self-perceptions of ageing, lower self-esteem and subjective age mediated the association of perceived age discrimination with depressive symptoms and poor wellbeing [25, 26]. Also, negative views on ageing regarding personal competence, physical decline, and social interactions were associated with perceived discrimination in the domains of work, medical care, and social life, respectively [13].

As a matter of fact, getting old is inevitable and is a coalition of various problems. Discrimination against older adults has come up as a social problem with the cusp of demographic and social structures. The extent of research on perceived age discrimination experienced every day by older adults is limited due to lack of high-quality evidence from large-scale national representative surveys of older adults in developing countries, specifically in India. Therefore, this study aims to examine the prevalence and correlates of perceived age discrimination among older adults using large nationally representative survey data in India.

## Methods

### Data

Data for the present study were drawn from the first wave of Longitudinal Study of Ageing in India (LASI wave-1) conducted during 2017–18. The survey was conducted by the International Institute for Population Sciences (IIPS), Mumbai, in collaboration with Harvard T. H. Chan School of Public Health (HSPH) and the University of Southern California (USC) under the stewardship of the Ministry of Health and Family Welfare (MoHFW), Government of India. LASI is a nationally representative longitudinal study of ageing and health that also covers the economic and social aspects of population ageing in India. The multistage stratified area probability cluster sampling method was used to select the sample. Within each state, a three-stage sampling design in rural areas and a four-stage sampling design in urban areas was adopted in the LASI wave-1 [27]. The study covered a total sample of 72,250 individuals aged 45 years and above and their spouses, irrespective of their age. Of which, around 31,464 were older adults aged 60 years and above [27]. The data is collected from 35 states and union territories of India (excluding Sikkim). LASI is envisioned to be conducted every two years for the next 25 years. The number of targeted primary sampling units (PSUs) in a state was given proportionally to each sub-state area in the first step, the selection of PSUs (sub-districts or Tehsils/Talukas) (level 1 stratification). The PSUs were chosen using Probability Proportional to Size (PPS) sampling in each area, with the number of households in each PSU serving as the size measure. The second stage entailed selecting a predetermined number of secondary sampling units (SSUs) from the selected PSUs, which are villages in rural regions and wards in urban areas. The third step in rural regions entailed selecting a number of households (HHs) (i.e. 32) from each designated village or village segment (for villages with more than 500 HHs). In metropolitan regions, the fourth round of selection entailed selecting a number of HHs (35 in this case) from each Census enumeration block (CEB). The interviews were conducted using computer-assisted personal interview (CAPI). The sample included for the present study was 31,464 older adults aged 60 and above.

### Outcome variable

The outcome variable used in the study was the perceived age discrimination based on a set of questions asked to the respondents. First, respondents were asked how often the below-listed things have happened to them in their day-to-day life: 1. you were treated with less courtesy or respect than other people; 2. received poorer service than other people at restaurants or stores; 3. people act as if they think you are not smart; 4. people act as if they are afraid of you; 5. threatened or harassed; 6. receive poorer service or treatment than other people from doctors or hospitals. The possible response options were recorded on 1 (almost everyday) to 6 (never) scale. Further, they were asked about the perceived reasons for such discrimination that included: age, gender, religion, caste, weight, physical disability, physical appearance, financial status and other reasons. Respondents who reported any experience of discrimination related to their age were used as sample of perceived age discrimination. Others, including those who did not experience any discrimination and experienced discrimination on other reasons excluding age, were considered as sample of not perceived as age discrimination. Perceived age discrimination takes the value ‘1’ if the respondent reported ‘yes’ otherwise, it takes the value ‘0’ representing no.

### Explanatory variables

Respondents' age was recoded into three categories: 60–69, 70–79, 80 years and above. Gender was categorized as male and female. Marital status was recoded as currently in marital union, widowed and, currently not in marital union (divorced/separated/deserted/live in relationship/never married) [28]. Educational attainment was classified as no education, 1–5 years, 5–10 years and, more than 10 years of education. Living arrangement was categorized as living with spouse and children, living with children and others, living with spouse and others and living alone. Social participation (member of any social organizations, religious groups, clubs, or societies) was coded as no and yes. Working status was categorized as never worked, earlier worked but currently not working and currently working. Residence was coded as rural and urban. Monthly per capita consumption expenditure (MPCE) quintile was classified as poorest, poor, middle, rich and richest. Caste was categorized as Other Backward Classes (OBC), Schedule castes and Schedule Tribes (SC/STs) and General (other than OBC/SC/ST). Religion was classified as Hindu, Muslim and others.

This study included four health measures i.e., self-rated general health (SRH), ability to do activity of daily life (ADL), ability to do instrumental activities of daily living (IADL) and chronic condition. SRH had a scale of 1 to 5 from “very good” to “very poor” and was categorized as 0 as good (representing very good, good and fair) and 1 as poor (representing poor and very poor) [29]. To quantify ADLs, respondents were asked, “Have you any difficulties in dressing, walking, bathing, eating, mobility and toilet?” A composite index was constructed from the questions mentioned above. The response variable “difficulty in ADL” was described as 0 as “no” and 1 as “yes” [30, 31]. The Cronbach’s alpha value for ADL scale was 0.869. To quantify IADLs, respondents were asked, “Have you any difficulties in preparing meal, shopping, making telephone, medication, doing work in garden or home, money handling and getting around?” A composite index was constructed from the questions mentioned above. The response variable “difficulty in IADL” was described as 0 as “no” and 1 as “yes” [31]. The Cronbach’s alpha value for IADL scale was 0.879. Respondents were asked about nine chronic conditions and one composite index was calculated to measure chronic conditions. Further, response variable 'chronic condition' was categorized into three categories: 0 as 'no chronic condition', 1 as 'having one condition', 2 as 'having 2 or more chronic conditions'.

### Statistical analysis

Univariate, bivariate and multivariable analyses have been conducted to examine the prevalence and factors associated with perceived age discrimination among older adults in India. Initially, descriptive statistics were performed to describe the variables of interest. Next, bivariate analysis with a chi-square test was employed to investigate the association of various socio-demographic and health-related factors with the perceived age discrimination. Further, a multivariable logistic regression model was used to determine the significant predictors of perceived age discrimination. The odds ratios of experiencing perceived age discrimination are reported by adjusting for various socio-economic covariates. Variance inflation factor was estimated to measure the multicollinearity among the variables used [32]. All the statistical analysis was performed using STATA-14.2. Additionally, the weights were applied which make the results nationally representative.

## Results

Table 1 represents the socio-economic and health profile of the study population. A proportion of 58.51% of respondents belonged to the 60–69 year age-group. The share of women respondents (52.55%) was higher than men (47.45%) in the total sample. More than one-third of older adults widowed. More than half of the older adults had no formal education. A proportion of 20.33% and 5.68% of older adults lived with spouses and alone, respectively. A substantially smaller share of older adults (4.69%) participated in social activities. Nearly one-third of older adults were currently working. About one-fourth of older adults were reported their general health status as poor. Nearly 23.59% and 47.97% of older adults reported difficulties in ADLs and IADLs, respectively. About 24% older adults had two or more chronic conditions. The majority of the study population (70.55%) resided in rural areas and 82.22% of participants belonged to the Hindu religion. A proportion of 16.73% older adults had perceived any kind of discrimination in their day-to-day life.

**Table 1:** Socio-economic and health profile of the study population (*N* = 31,464)

Characteristics	Frequency (*N*)	Percent
**Age**		
60–69	18,974	58.51
70–79	9,101	30.2
80 and above	3,389	11.29
**Gender**		
Male	15,098	47.45
Female	16,366	52.55
**Marital Status**		
Currently in marital union	19,920	61.63
Widowed	10,719	36.2
Currently not in marital union	825	2.17
**Years of schooling**		
No	16,889	56.52
1–5 years	5,840	17.5
5–10 years	6,106	18.24
More than 10 years	2,629	7.74
**Living arrangement**		
Living with spouse and children	13,465	40.62
Living with children and others	10,162	33.37
Living with spouse	6,215	20.33
Living alone	1,622	5.68
**Social participation**		
No	28,885	95.31
Yes	2,128	4.69
**Work status**		
Never worked	8,784	26.43
Earlier worked	13,373	42.81
Currently working	9,307	30.76
**Self rated health**		
Good	23,685	73.78
Poor	7,779	26.22
**Difficulties in ADL**		
No	24,770	76.41
Yes	6,694	23.59
**Difficulties in IADL**		
No	17,609	52.03
Yes	13,855	47.97
**Chronic Condition**		
No	14,335	46.62
One	9,241	29.33
Two and more	7,797	24.05
**Residence**		
Urban	10,739	29.45
Rural	20,725	70.55
**MPCE quintile**		
Poorest	6,484	21.7
Poor	6,477	21.71
Middle	6,416	20.95
Rich	6,170	19.19
Richest	5,917	16.45
**Caste**		
General	9,265	27.74
OBC	11,886	45.23
SC/ST	10,313	27.03
**Religion**		
Hindu	23,037	82.22
Muslim	3,731	11.28
Others	4,696	6.5
**Region**		
Northern Region	5,171	11.83
North-Eastern Region	3,752	2.97
Eastern Region	6,280	23.66
Central Region	4,903	21.71
Southern Region	11,358	39.83
**Facing any kind of discrimination**		
No	26,864	83.27
Yes	4,600	16.73

*ADL* Activities of daily living, *IADL* Instrumental activities of daily living, *OBC* Other backward class, *SC/ST* Scheduled caste/ Scheduled tribe, *MPCE* Monthly per capita consumption expenditure

**Fig. 1:**
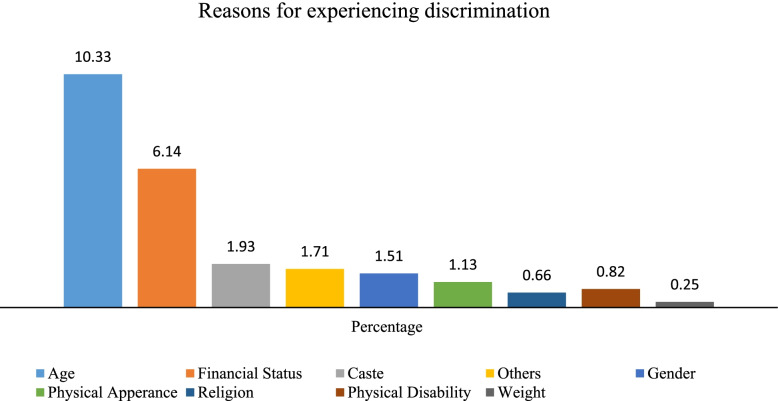
Percentage of older adults who perceived discrimination by major reasons, LASI, 2017–18 (*N* = 31.464)

Figure 1 shows the reasons for experiencing discrimination among older adults. About one-tenth of older adults perceived their age as the main reason for discrimination. About 6.14% of older adults perceived their financial status as the main reason behind discrimination in old age, whereas 1.93% and 1.51% older adults reported perceived discrimination on the basis of their caste affiliation and gender, respectively.

Table 2 represents the prevalence of perceived age discrimination among older adults by their background characteristics. Perceived age discrimination was seen to be higher in older age groups which rose to 11.86% among oldest-old people aged 80 and above. The prevalence of perceived age discrimination was slightly higher among females (10.86%) and those residing in rural areas (11.36%) as compared to their male counterparts (9.74%) and those living in urban areas (7.85%), respectively. The perceived age discrimination was also seen to be more common among those currently not in a marital union or widowed. About 12% of the respondents with no formal education reported perceived age discrimination. Older adults who lived alone had the highest prevalence of perceived age discrimination (16.58%). Also, older adults who earlier worked but currently not working had a higher prevalence of perceived age discrimination. Older adults who reported poor self-rated health (10.89%), difficulties in ADL (11.74%), difficulties in IADL (12.49%), having one chronic condition (10.84%) had higher prevalence of perceived age discrimination. Perceived age discrimination was highest in the poorest MPCE quintile (12.55%).

**Table 2:** Prevalence of perceived age discrimination among older adults by background characteristics, LASI, 2017–18 (*N* = 31,464)

Characteristics	No perceived age discrimination n(%)	Perceived age discrimination n(%)	*p* value
**Age (in years)**			< 0.001
60–69	17,319 (90.16)	1655 (9.84)	
70–79	8147 (89.3)	954 (10.7)	
80 and above	3000 (88.14)	389 (11.86)	
**Gender**			0.001
Male	13,748 (90.26)	1350 (9.74)	
Female	14,718 (89.14)	1648 (10.86)	
**Marital Status**			< 0.001
Currently in marital union	18,221 (90.67)	1699 (9.33)	
Widowed	9508 (88.1)	1211 (11.9)	
Currently not in marital union	737 (87.58)	88 (12.42)	
**Years of schooling**			< 0.001
No	15,019 (88.15)	1870 (11.85)	
1–5 years	5312 (89.66)	528 (10.34)	
5–10 years	5683 (92.83)	423 (7.17)	
More than 10 years	2452 (93.34)	177 (6.66)	
**Living arrangement**			< 0.001
Living with spouse and children	12,375 (90.94)	1090 (9.06)	
Living with children and others	9079 (88.87)	1083 (11.13)	
Living with spouse	5634 (90.2)	581 (9.8)	
Living alone	1378 (83.42)	244 (16.58)	
**Social participation**			< 0.001
No	26,037 (89.37)	2848 (10.63)	
Yes	1979 (91.53)	149 (8.47)	
**Work status**			< 0.001
Never worked	8051 (92.28)	733 (7.72)	
Earlier worked	11,928 (88.05)	1445 (11.95)	
Currently working	8487 (89.69)	820 (10.31)	
**Self rated health**			< 0.001
Good	21,526 (89.87)	2159 (10.13)	
Poor	6940 (89.11)	839 (10.89)	
**Difficulties in ADL**			< 0.001
No	22,621 (90.11)	2149 (9.89)	
Yes	5845 (88.26)	849 (11.74)	
**Difficulties in IADL**			< 0.001
No	16,321 (91.66)	1288 (8.34)	
Yes	12,145 (87.51)	1710 (12.49)	
**Chronic Condition**			0.383
No	12,994 (89.56)	1341 (10.44)	
One	8327 (89.16)	914 (10.84)	
Two and more	7058 (90.3)	739 (9.7)	
**Residence**			< 0.001
Urban	9886 (92.15)	853 (7.85)	
Rural	18,580 (88.64)	2145 (11.36)	
**MPCE quintile**			< 0.001
Poorest	5770 (87.45)	714 (12.55)	
Poor	5881 (89.59)	596 (10.41)	
Middle	5838 (90.92)	578 (9.08)	
Rich	5594 (90.04)	576 (9.96)	
Richest	5383 (90.69)	534 (9.31)	
**Caste**			< 0.001
General	8488 (91.14)	777 (8.86)	
OBC	10,586 (89.33)	1300 (10.67)	
SC/ST	9392 (88.73)	921 (11.27)	
**Religion**			< 0.001
Hindu	20,601 (89.11)	2436 (10.89)	
Muslim	3450 (91.03)	281 (8.97)	
Others	4415 (94.41)	281 (5.59)	
**Region**			< 0.001
Northern Region	4726 (90.99)	445 (9.01)	
North-Eastern Region	3509 (93.17)	243 (6.83)	
Eastern Region	5848 (92.11)	432 (7.89)	
Central Region	4081 (83.74)	822 (16.26)	
Southern Region	10,302 (90.81)	1056 (9.19)	
**Total**	**28,466 (89.67)**	**2994 (10.33)**	

*ADL* Activities of daily living, *IADL* Instrumental activities of daily living, *OBC* Other backward class, *SC/ST* Scheduled caste/ Scheduled tribe, *MPCE* Monthly per capita consumption expenditure

Table 3 depicts the multivariable logistic regression estimates for perceived age discrimination among older adults. The odds of perceived age discrimination increased with increasing age. Females were more likely to perceive age discrimination than their male counterparts [AOR: 1.12; CI: 0.95—1.32]. Older adults who were not in the marital union or widowed had a positive association with perceived age discrimination than currently married older adults. The odds of perceived age discrimination decreased with an increase in educational attainment. Older adults with more than 10 years of schooling were 32% [AOR: 0.68; CI: 0.51—0.89] less likely to perceive age discrimination. Older adults who earlier worked but currently not working were 73% [AOR: 1.73; CI: 1.46 – 2.05] more likely to have perceived age discrimination than those who never worked. Older adults having difficulties in IADL [AOR: 1.43; CI: 1.25 -1.65] and having one chronic condition [AOR: 1.16; CI: 1.02—1.34] had a positive association with perceived age discrimination. Rural resident older adults were 17% [AOR: 1.17; CI: 1.01 – 1.35] more likely to perceive age discrimination than their urban counterparts. Additionally, the odds of perceived age discrimination were lower among the richest MPCE quintile [AOR: 0.80; CI:0.66—0.97] than those in the poorest MPCE quintile. Older adults from the central part of the country had the highest odds of perceived age discrimination [AOR: 1.72; CI:1.43—2.07] compared to older adults from the northern part of the country.

**Table 3:** Multivariable logistic regression estimates for perceived age discrimination by background characteristics among older adults, LASI, 2017–18 (*N* = 31,464)

Covariates	AOR (95% CI)
**Age**	
60–69®	
70–79	1.01 (0.86—1.16)
80 and above	1.02 (0.83—1.26)
**Gender**	
Male®	
Female	1.12 (0.95—1.32)
**Marital Status**	
Currently in marital union®	
Widowed	1.03 (0.5—2.13)
Currently not in marital union	1.13 (0.5—2.55)
**Years of schooling**	
No®	
1–5 years	0.97 (0.82—1.14)
5–10 years	0.78**(0.64—0.95)
More than 10 years	0.68***(0.51—0.89)
**Living arrangement**	
Living with spouse and children®	
Living with children and others	1.12 (0.54—2.32)
Living with spouse	1.11 (0.94—1.31)
Living alone	1.69 (0.81—3.55)
**Social participation**	
No®	
Yes	1.02 (0.79—1.32)
**Work status**	
Never worked®	
Earlier worked	1.73***(1.46—2.05)
Currently working	1.61***(1.31—1.96)
**Self rated health**	
Good®	
Poor	0.94 (0.82—1.07)
**Difficulties in ADL**	
No®	
Yes	1.02 (0.87—1.18)
**Difficulties in IADL**	
No®	
Yes	1.43***(1.25—1.65)
**Chronic Condition**	
No®	
One	1.16**(1.02—1.34)
Two and more	1.17*(0.99—1.39)
**Residence**	
Urban®	
Rural	1.17**(1.01—1.35)
**MPCE quintile**	
Poorest®	
Poor	0.84**(0.7—0.99)
Middle	0.76***(0.64—0.9)
Rich	0.82*(0.68—1.01)
Richest	0.8**(0.66—0.97)
**Caste**	
General®	
OBC	1.03 (0.89—1.21)
SC/ST	1.02 (0.84—1.23)
**Religion**	
Hindu®	
Muslim	0.89 (0.69—1.15)
Others	0.56***(0.43—0.72)
**Region**	
Northern Region®	
North-Eastern Region	0.77**(0.59—0.99)
Eastern Region	0.75***(0.62—0.91)
Central Region	1.72***(1.43—2.07)
Southern Region	0.88 (0.73—1.05)

*ADL* Activities of daily living, *IADL* Instrumental activities of daily living, *OBC* Other backward class, *SC/ST* Scheduled caste/ Scheduled tribe, *MPCE* Monthly per capita consumption expenditure

^*^p < 0.1, **p < .05, ***p < .01; ®- Reference category

*AOR* Odds Ratio adjusted for all the covariates in the study

## Discussion

Perceived discrimination because of one’s age is an important social and public health issue that affects the physical and mental health and civil rights of older people worldwide. Older people are at higher risk of social exclusion, loneliness and abandonment due to their various health conditions and discriminatory behaviour of the general public [24]. With the increasing number of older people in India, this issue deserves a greater attention for society, government and policy-makers. Hence, the present study is an attempt to understand the prevalence and correlates of perceived age discrimination among older adults using large nationally representative survey data.

The level of perceived age discrimination is lower (10.33%) compared to available evidence from the western world. A study using data from the Health and Retirement Study in the United States found that 30% of respondents aged 50 and above perceived age as the most common reason for discrimination in their day to day life [18]. Rippon et al. (2014) reported prevalence of age discrimination was 33.3% among respondents aged 52 years and above [17]. The possible explanation for lower prevalence could be the absence of uniform reporting and understanding of discrimination and lack of knowledge. In accordance with previous studies [17, 18, 33, 34], we found that rate of perceived age discrimination increased with age. The percentages are higher among adults in the oldest old age category, with a rate of 11.86 percent. Although respect for older people is strongly embedded in family and social life in India and other Asian countries [35], recent studies suggest a transition in the general behaviour and an increase in negative attitudes towards older people in households and community settings in India [36, 37]. Increased institutionalisation of older persons, which is associated with social and emotional losses, also indicates the decline in respect to the senior members of the society [38]. Again, as evidence suggests, the detrimental effects of perceived age discrimination would be particularly pronounced in the case of adults in their later years because they belong to a lower status group [24].

The findings suggest that a variety of socio-demographic, economic and health variables precede a greater likelihood of perceived age discrimination. This supports the claim that one’s mood impacts the interpretation of everyday events which could potentially expose older adults to higher levels of perceived age discrimination [39, 40]. Older individuals often interpret these mood-related issues as signs of old age and respond negatively to them which is termed as ‘healthism’ [41]. In agreement with this, health-related variables such as difficulty in instrumental activities of daily living and higher number of chronic conditions show significant associations with perceived age discrimination among the older individuals in the present study. Further, the significant association of co-residential living arrangements and currently being in a marital union with lower levels of perceived age discrimination are consistent with past studies [42, 43]. Again, although it was statistically insignificant in multivariable analysis, bivariate results show that in line with past studies, lack of social participation increased the prevalence of perceiving age discrimination among older population [44–46]. This supports the notion related to social disengagement theory which suggests that older people withdraw themselves from all sorts of involvement in the social activities and if they want to connect to the companionship they formerly enjoyed, the opportunities will be limited for several reasons [47].

Our findings also indicate that respondents who earlier worked or currently working perceived age discrimination more than those who never worked. This is inconsistent with findings of the studies in developed countries [25], which found that work status was a strong predictor of perceptions of age discrimination, with a larger proportion of older individuals who were unemployed reporting age discrimination in comparison to those who were working. This might partially be explained by the fact that people at the workplace may be more aware of the consequences of such discrimination and avoid in their interactions with older co-workers. Further, the results of the study revealed negative associations between perceived age discrimination and the two indicators of household wealth and education with those educated and wealthy people having less likelihood of perceived age discrimination. The possible explanation could be greater awareness regarding social policy and rights of older persons in the higher socioeconomic groups and sources of resilience to such social disorders [16, 46, 48]. Thus, the results suggest that discrimination or at least perceived age discrimination is commonly experienced by those who have disadvantaged socioeconomic statuses in terms of education and wealth.

Some limitations of this study need to be acknowledged. The data were drawn from self-report, potentially creating response biases and limiting the validity of the measures of perceived age discrimination. Differences in objective versus perceived discrimination could exist for multiple reasons. In the same vein, perceived discrimination is not synonymous with actual discrimination. Findings might have been different had we examined actual occurrences of discrimination. Also, the co-occurrence between perceived age discrimination and discrimination due to different reasons needs to be explored. The survey included information about other reasons for discrimination; however, in order to make it a more focused investigation on age discrimination, other reasons could not be explored. Despite these limitations, the study utilizes a large nationally representative sample of the older adult population which gives more generalizability of the findings. Also, a variety of health outcomes associated with perceived age discrimination is provided that add to the existing literature on the wellbeing of an ageing population.

## Conclusion

Older adults with lower socioeconomic status, currently working, having more chronic conditions, difficulty in IADL and belonging to rural areas were found to perceive higher age discrimination than their counterparts. The findings of the study have important implications for policy makers with respect to strategies such as making the vulnerable populations aware of their legal rights that help in the prevention of age-based discrimination in the country. India lacks a robust policy and legal provision to prevent such incidents, which is the need of the hour to be addressed. The discrimination faced by older people cannot resolve from a single authorization. There is a need for special measures at family, institutions, community and government levels to eliminate day to day discrimination against older people. Aged people should be considered as an asset for the country as they have more experience, knowledge and wisdom than their younger counterparts. There should be changes in negative attitude towards aged people at the community level. Also, Health providers should be aware that discrimination is a major stressor in old age, especially among vulnerable populations with poor socioeconomic and health status.

## Data Availability

The study uses secondary data which is available on reasonable request through https://www.iipsindia.ac.in/content/lasi-wave-i.

## Abbreviations

AOR: Adjusted odds ratio
CI: Confidence interval
ADL: Activities of daily living
IADL: Instrumental activities of daily living
MPCE: Monthly per capita consumption expenditure

## References

[1] U.N. World population ageing, 2015 report (2015). United Nations, department of economic and social affairs, population division.

[2] United Nations. Department of Economic and Social Affairs Population Division. World Population Ageing 2017 report. Geneva; 2017.

[3] Bloom DE, Canning D, Fink G. Implications of population ageing for economic growth. Oxford review of economic policy. 2010;26(4):583–612.

[4] Canning D (2011). The causes and consequences of demographic transition. Popul Stud (NY).

[5] Véron J. National research Council—Preparing for an aging world: the case for cross. National Research. Population. 2001;56(5):885–6.

[6] Rychtařiková J. Perception of population ageing and age discrimination across EU countries. Population and Economics. 2019;3:1.

[7] American Psychological Association. Stress in America: The Impact of Discrimination. Stress in America Survey. Washington, DC; 2016.

[8] Andriessen I, Dagevos J (2014). Disadvantages in the labor market for ethnic minority men and women. Racism: global perspectives, coping strategies and social implications.

[9] Palmore EB. Journal of Gerontology : Ageism Comes of Age. J Gerontol Soc Sci. 2015;70(6):873–5. 10.1093/geronb/gbv079 PMC4851714 26362603

[10] Chang ES, Kannoth S, Levy S, Wang SY, Lee JE, Levy BR. Global reach of ageism on older persons’ health: A systematic review. PloS one. 2020;15(1):e0220857. 10.1371/journal.pone.0220857 PMC6961830 31940338

[11] Nelson TD (2015). Handbook of prejudice, stereotyping, and discrimination: second edition.

[12] Nelson SH. Shunned: Discrimination Against People With Mental Illness. 2007:19551.

[13] Voss P, Wolff JK, Rothermund K (2017). Relations between views on ageing and perceived age discrimination: a domain-specific perspective. Eur J Ageing.

[14] Voss P, Bodner E, Rothermund K (2018). Ageism: the relationship between age stereotypes and age discrimination.

[15] Gee G, Walsemann K. Does health predict the reporting of racial discrimination or do reports of discrimination predict health? Findings from the National Longitudinal Study of Youth. Soc Sci Med. 2009;68(9):1676–84. 10.1016/j.socscimed.2009.02.002 19289253

[16] Gee GC, Pavalko EK, Long JS (2007). Age, cohort and perceived age discrimination: Using the life course to assess self-reported age discrimination. Soc Forces.

[17] Rippon I, Kneale D, de Oliveira C, Demakakos P, Steptoe A. Perceived age discrimination in older adults. Age Ageing. 2014;43(3):379–86. 10.1093/ageing/aft146 PMC4081784 24077751

[18] Luo Y, Xu J, Granberg E, Wentworth WM (2012). A longitudinal study of social status, perceived discrimination, and physical and emotional health among older adults. Res Aging.

[19] Krieger N. Discrimination and health inequities. Int J Health Serv. 2014;44(4):643–710. 10.2190/HS.44.4.b 25626224

[20] Jackson SE, Hackett RA, Steptoe A (2019). Associations between age discrimination and health and wellbeing: cross-sectional and prospective analysis of the english longitudinal study of ageing. Lancet Public Heal.

[21] Flores E, Tschann JM, Dimas JM, Bachen EA, Pasch LA, De Groat CL (2008). Perceived discrimination, perceived stress, and mental and physical health among Mexican-origin adults. Hisp J Behav Sci.

[22] Taylor J, Turner RJ. Perceived discrimination, social stress, and depression in the transition to adulthood : racial contrasts. Soc Psychol Q. 2002;65(3):213–25.

[23] Vogt Yuan AS. Perceived age discrimination and mental health. Soc Forces. 2007;86(1):291–311.

[24] Garstka TA, Schmitt MT, Branscombe NR, Hummert ML (2004). How young and older adults differ in their responses to perceived age discrimination. Psychol Aging.

[25] Han J, Richardson VE (2015). The relationships among perceived discrimination, self-perceptions of aging, and depressive symptoms: a longitudinal examination of age discrimination. Aging Ment Heal.

[26] Marquet M, Chasteen AL, Plaks JE, Balasubramaniam L (2019). Understanding the mechanisms underlying the effects of negative age stereotypes and perceived age discrimination on older adults’ well-being. Aging Ment Heal.

[27] International Institute for Population Sciences (IIPS), NPHCE, MoHFW HTHCS of PH (HSPH) and the U of SC (USC). 2020. Longitudinal Ageing Study in India ( LASI ) Wave 1, 2017–18, India Report. Mumbai; 2020.

[28] Srivastava S, Joseph KJV, Dristhi D, Muhammad T (2021). Interaction of physical activity on the association of obesity-related measures with multimorbidity among older adults: a population-based cross-sectional study in India. BMJ Open.

[29] Srivastava S, Muhammad T (2020). Violence and associated health outcomes among older adults in India: a gendered perspective. SSM - Popul Heal.

[30] Muhammad T, Maurya P, Sharma P (2021). Prevalence and correlates of bone and joint diseases and its association with falls among older adults in India: evidence from LASI, 2017–18. Geriatr Nurs (Minneap).

[31] Muhammad T, Meher T (2021). Association of late-life depression with cognitive impairment: evidence from a cross-sectional study among older adults in India. BMC Geriatr.

[32] Lewis-Beck M, Bryman A, Liao TF (2004). Variance inflation factors. The SAGE encyclopedia of social science research methods.

[33] Avidor S, Ayalon L, Palgi Y, Bodner E (2017). Longitudinal associations between perceived age discrimination and subjective well-being: variations by age and subjective life expectancy. Aging Ment Heal.

[34] Ayalon L, Gum AM (2011). The relationships between major lifetime discrimination, everyday discrimination, and mental health in three racial and ethnic groups of older adults. Aging Ment Heal.

[35] Ingersoll-Dayton B, Saengtienchai C (1999). Respect for the elderly in Asia: Stability and change. Int J Aging Hum Dev.

[36] Parkar SR (2015). Elderly mental health: Needs. Mens Sana Monogr.

[37] Srivastava S, Singh SK, Kumar M, Muhammad T (2021). Distinguishing between household headship with and without power and its association with subjective well-being among older adults: an analytical cross-sectional study in India. BMC Geriatr.

[38] Sarin K, Punyaapriya P, Sethi S, Nagar I. Depression and hopelessness in institutionalized elderly: a societal concern. Open J Depress. 2016;05(03):21–7.

[39] Kydd A, Fleming A (2017). Ageism in the third age. Innovation in Aging.

[40] Lee Y, Bierman A (2018). A Longitudinal assessment of perceived discrimination and maladaptive expressions of anger among older adults: does subjective social power buffer the association?. J Gerontol B Psychol Sci Soc Sci.

[41] Roberts JL, Leonard EW. What is (and Isn't) Healthism. Ga. L. Rev.. 2015;50:833.

[42] Vogt Yuan AS (2007). Perceived age discrimination and mental health. Soc Forces.

[43] Van Den Heuvel WJA (2012). Discrimination against older people. Rev Clin Gerontol.

[44] Hooker K, Mejía ST, Phibbs S, Tan EJ, Stevens J (2019). Effects of age discrimination on self-perceptions of aging and cancer risk behaviors. Gerontologist.

[45] McGann M, Ong R, Bowman D, Duncan A, Kimberley H, Biggs S (2016). Gendered ageism in Australia: changing perceptions of age discrimination among older men and women. Econ Pap.

[46] Giasson HL, Queen TL, Larkina M, Smith J (2017). Age group differences in perceived age discrimination: associations with self-perceptions of aging. Gerontologist.

[47] Islam MR (2016). Ageism and age discrimination in old age: an overview. Philos Prog.

[48] Kessler R, Mickelson K, Williams D (1999). The prevalence, distribution, and mental health correlates of perceived discrimination in the United States. J Health Soc Behav.

